# How to Dispose of Medical Waste Caused by COVID-19? A Case Study of China

**DOI:** 10.3390/ijerph182212127

**Published:** 2021-11-19

**Authors:** Min Su, Qiang Wang, Rongrong Li

**Affiliations:** 1 School of Economics and Management, China University of Petroleum (East China), Qingdao 266580, China; B19080004@s.upc.edu.cn (M.S.); wangqiang7@upc.edu.cn (Q.W.); 2 Institute for Energy Economics and Policy, China University of Petroleum (East China), Qingdao 266580, China

**Keywords:** COVID-19, coronavirus, medical waste, emergency disposal, China

## Abstract

The rapid increase in novel coronavirus (COVID-19) patients also means a rapid increase in medical waste that could carry the novel coronavirus (SARS-CoV-2). How to safely dispose of medical waste caused by COVID-19 is a huge challenge that needs to be solved urgently. The outbreak of the COVID-19 has led to a significant increase in the daily generation of medical waste in China and has placed a severe test on the Chinese medical waste disposal system. Unlike ordinary wastes and garbage, medical waste that is untreated or incompletely treated will not only cause environmental pollution, but also directly or indirectly cause infections and endanger people’s health. Faced with difficulties, the Chinese government formulated a policy for medical waste management and a response plan for the epidemic, which provides policy guarantee for the standardized disposal of epidemic medical waste. In addition, the government and medical institutions at all levels formed a comprehensive, refined, and standardized medical treatment process system during research and practice. China has increased the capacity of medical waste disposal in various places by constructing new centralized disposal centers and adding mobile disposal facilities. China has achieved good results in the fight against COVID-19, and the pressure on medical waste disposal has been relieved to a certain extent. However, the global epidemic situation is severe. How to ensure the proper and safe disposal of medical waste is related to the prevention and control of the epidemic situation. This study summarizes China’s experience in the disposal of medical waste in the special case of COVID-19 and hopes to provide some reference for other countries in the disposal of medical waste.

## 1. Introduction

The 2019 novel coronavirus disease (COVID-19) is a highly contagious disease caused by new coronavirus infection (severe acute respiratory syndrome coronavirus 2, SARS-CoV-2) [1]. In December 2019, COVID-19 was first reported in Wuhan, Hubei Province, China, and then broke out throughout the country and even the world [2,3]. The outbreak and spread of COVID-19 worldwide has seriously threatened public health [3,4].

During the COVID-19 epidemic, the daily generation of medical waste increased significantly, placing a severe test on China’s medical waste disposal system [5]. The diagnosis and treatment of COVID-19 patients not only produced conventional medical waste, but also produced contaminated protective clothing, masks, gloves, and other protective equipment. In addition, there is also patients’ household garbage. This has led to a surge in the total amount of medical waste and increased pressure on medical waste disposal [6]. Unlike ordinary wastes and garbage, medical waste that is untreated or incompletely treated will not only cause environmental pollution, but also directly or indirectly cause infections and endanger people’s health [7,8,9].

Faced with difficulties, the Chinese government has formulated medical waste management policies and epidemic response plans, which provides policy guarantee for the standardized disposal of epidemic medical waste [10,11]. From 20 January to 11 April, China accumulatively disposed of 256,000 tons of medical waste. As of 11 April, the national daily disposal capacity of medical waste was 6062.4 tons, an average daily increase of 1159.6 tons compared with 4902.8 tons before the epidemic.

China has achieved good results in the fight against COVID-19 [12], and the pressure on medical waste disposal has been relieved, to a certain extent [13,14]. However, the global epidemic situation is severe. As of 24:00 on 15 April 2020, a total of 1,964,021 cases were diagnosed globally [15]. Ensuring the proper and safe disposal of medical waste is related to the prevention and control of epidemic situation [16]. This study summarizes China’s experience in the disposal of medical waste in the special case of COVID-19 and hopes to provide some reference for other countries in the disposal of medical waste.

## 2. Background Information

**Figure 1:**
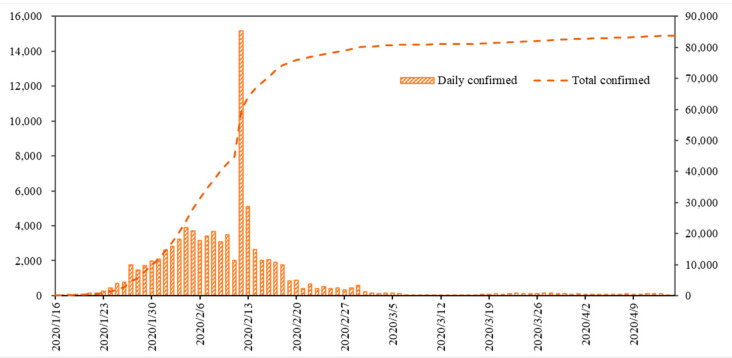
The trend of daily confirmed cases of COVID-19 in China (vertical axis: person; horizontal axis: time) (data source: National Health Commission of the People’s Republic of China. Daily briefing on novel coronavirus cases in China, http://en.nhc.gov.cn/DailyBriefing.html (accessed on 16 April 2020)).

Starting in December 2019, Wuhan, Hubei Province, China reported a series of unexplained cases of pneumonia [17,18]. On 7 January 2020, through deep sequencing analysis of the patient’s respiratory tract, China identified a new coronavirus (SARS-CoV-2; previously known as 2019-nCoV) as pathogenic bacteria [19]. Subsequently, COVID-19 quickly spread to other parts of Hubei Province and nationwide, seriously threatening public health [20,21]. Since 18 January, the epidemic has spread rapidly throughout China due to large-scale migration related to the Chinese New Year. By 29 January, confirmed cases were recorded in all provinces and regions of China [22,23]. As of 24:00 on 15 April 2020, according to the National Health Commission of the People’s Republic of China, 83,797 cases were reported nationwide. Among them, 67,803 cases and 50,008 cases were diagnosed in Hubei Province and Wuhan City, accounting for 80.91% and 59.68% of the country, respectively. Figure 1 shows the trend of daily confirmed cases. From 16 January to 3 February, the number of daily confirmed cases increased rapidly. From 4 February to 15 April, the number of daily confirmed cases showed a general downward trend. Among them, the number of daily confirmed cases reached 15,153 on 12 February, becoming the day with the largest number of daily confirmed cases. On March 7, the number of daily confirmed cases fell below 100 for the first time. From 7 March, the number of daily confirmed cases is less than 150 and is at a stable level. This shows that China’s battle against the COVID-19 epidemic has achieved significant results.

## 3. Medical Waste

**Figure 2:**
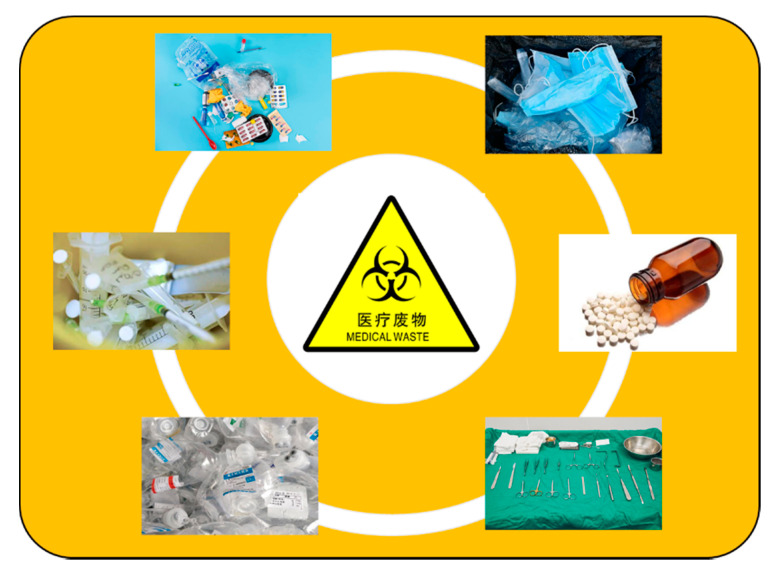
Medical waste. From [26,27].

According to the “Medical Waste Management Regulations” (MWMR) promulgated by the State Council of China in 2003, medical waste refers to wastes with direct or indirect infectiousness, toxicity, and other hazards generated by medical institutions in medical treatment, prevention, health care, and other related activities [24]. In the same year, the Ministry of Health and the State Environmental Protection Administration formulated the “Classified Catalogue of Medical Waste” based on the MWMR, which divided medical waste into five categories, namely, infectious waste, pathological waste, toxic waste, medical waste, and chemical waste (Table 1). Generally speaking, medical waste is considered as special hazardous waste with complex composition [25]. Therefore, standardizing the management and disposal of medical waste is essential to prevent the spread of diseases and protect public safety. Figure 2 illustrates common medical waste.

Medical waste has the characteristics of strong toxicity, strong corrosiveness, and strong pathogenicity [29]. If it is improperly disposed, it will not only severely damage the ecological environment, but also pose a threat to human health [30,31,32]. Random disposal of medical waste is likely to contaminate the soil and reduce the available land resources, because medical waste usually contains heavy metals and synthetic organics, which are harmful to human health and are difficult to degrade. When toxic medical waste is piled in the open air, the harmful substances in it will flow into rivers, lakes, and oceans with rainwater, causing serious water pollution [33]. The deterioration of water quality will directly affect the safety of drinking water and endanger human health. Moreover, water pollution will adversely affect the normal growth of aquatic organisms, thereby destroying the ecological balance of the water environment [34,35]. More importantly, once the infectious pathogens in medical waste enter the water body, it will cause the rapid spread of infectious diseases [36].

**Figure 3:**
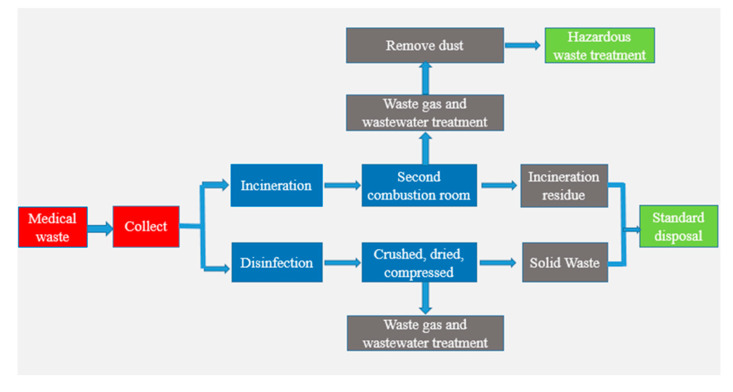
The basic process of medical waste disposal. From [41].

On the other hand, improper disposal of medical waste will also pollute the air and even release harmful gases. When the temperature and the appropriateness reach certain conditions, the organic matter in the medical waste will decompose and produce harmful gases and dust [37]. The diffusion of these harmful substances into the atmosphere will cause the deterioration of air quality, and, at the same time, endanger public health and ecological balance [38,39]. At present, China has formed a medical waste centralized treatment technology system focusing on ossification and disinfection [40]. Table 2 lists the commonly used medical waste treatment technologies and their scope of application in China. Figure 3 illustrates the basic flow of medical waste treatment.

## 4. Legal System for Medical Waste Disposal

### 4.1. Laws and Regulations on Medical Waste Disposal in China

**Figure 4:**
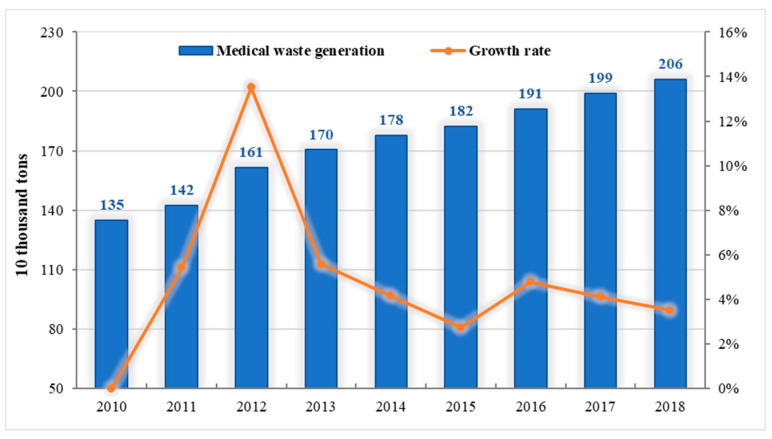
China medical waste generation and growth from 2010 to 2018. From [49].

In the process of rapid development of health care in our country, an alarming amount of medical waste is continuously produced. According to data from the National Bureau of Statistics, from 2010 to 2018, the amount of medical waste in China increased from 1.349 million tons to 2.0601 million tons (Figure 4), showing an upward trend year by year. If these medical wastes cannot be strictly and effectively controlled and managed, it is likely to cause environmental pollution and bring huge pressure to global environmental protection [42,43]. In this case, it is necessary to formulate and implement medical waste disposal laws and regulations. A scientific legal system helps to properly dispose of medical waste without damaging the ecological environment and endangering social security [44,45]. From Figure 4, although the total amount of medical waste increased year by year, the growth rate slowed down since 2012 and entered a slow growth stage. This trend reveals that the standardized management of medical waste in China has achieved certain results, which means that the medical waste management system is gradually improved. The establishment of the medical waste management system is inseparable from the support of policies [46,47,48]. Table 3 lists the main laws, regulations, and policies in China on medical waste disposal.

China promulgated a series of laws and regulations on medical waste disposal, which fully reflects the government’s emphasis on medical waste management [50]. After years of practical discussion and theoretical research, China has initially formed a relatively complete legal system for medical waste management. The system takes “The Constitution” as the basic law, and “The Environmental Protection Law”, “The Law on the Prevention and Treatment of Infectious Diseases”, and the MWMR as the main body. In fact, China’s medical waste management system follows the basic principles of centralization and harmlessness and classifies medical waste as hazardous waste for standardized management. These laws and regulations have made clear regulations on how to properly dispose of medical waste and proposed specific requirements for medical institutions and medical staff [51]. Their main content can be summarized as the following aspects:

(1) Medical institutions should establish a responsibility system for medical waste management. The legal representative of a medical institution shall be responsible for the medical waste generated by the institution, and promptly and correctly dispose of the medical waste to prevent the spread of infectious diseases and environmental pollution accidents.
(2) Medical institutions need to train their staff to improve their medical waste management awareness and medical waste disposal capabilities.
(3) The personnel and management personnel engaged in medical waste work in medical institutions shall be equipped with necessary protective equipment. At the same time, it is necessary to check the health status of relevant staff regularly to prevent them from suffering health damage.
(4) Medical institutions should promptly register medical wastes. The registration content includes source, type, weight, handover time, disposal method, final destination, signature of the handler, etc. Under normal circumstances, this information needs to be kept for at least 3 years.
(5) For medical wastes that may contain infectious viruses, medical institutions should take effective measures to concentrate on harmless treatment, with the purpose of preventing their loss, leakage, and spread, and adversely affecting public health and the ecological environment.
(6) If there are places, objects, and medical waste contaminated by infectious disease pathogens in medical institutions, the unit should be disinfected in time and the medical waste should be treated in a harmless manner.

### 4.2. Laws and Regulations on Medical Waste Disposal in Other Typical Countries

#### 4.2.1. USA

The United States is considered to be one of the countries with the most complete environmental laws and regulations system among the developed countries in the world. The U.S. medical waste policy system develops framework guidelines at the federal level and focuses on state management. In fact, in the 1970s, the United States Environmental Protection Agency (USEPA) began advocating and encouraging the use of incineration to treat medical waste. They believe that the incineration of potentially infectious medical waste can stop the spread of diseases. Although the United States continues to strengthen pollution control requirements for hazardous waste incineration, incomplete combustion produces a large amount of air pollutants, which has an adverse impact on human health and the ecological environment. Therefore, the United States began to pay attention to and standardize the management of medical waste. The earliest medical waste management regulations in the United States were promulgated in 1988, which was the “Medical Waste Tracking Act” (MWTA) enacted by the US Congress. The validity period of this regulation is two years after expiration; each state assumes the role of formulating medical waste management regulations. For example, California promulgated the “Medical Waste Management Act” (MWMA) in 2017 [52], which stipulates the management methods for the production, transportation, and disposal of medical waste. After the outbreak of COVID-19, according to the Medical Waste Management Program (MWMP), the state issued the “Novel Coronavirus Disease 2019 (COVID-19) Medical Waste Management Interim Guidelines”, which provides corresponding operation standards guidance on the generation, storage, transportation, and disposal of medical waste during COVID-19.

In the United States, there are corresponding management systems and tracking methods for medical waste from generation, classification, storage, and transportation to final disposal. Relevant departments have not only formulated a medical waste management plan, but also built a professional medical waste disposal site and assigned a professional person in charge in the city. At present, the medical waste management policies of the United States at the national level mainly include: “Resource Conservation and Recovery Act” (RCRA, 1976) [53], “Medical Waste Management Strategy” (MWMS, 1988), “Model Guidelines for State Medical Waste Management” (1992), “the Hospital Medical Infectious Waste Inhibitor” (HMIWI, 2013), etc. In general, the United States has established a relatively perfect medical waste management system and professional treatment process [54].

#### 4.2.2. United Kingdom

The “British Environmental Law” promulgated by the United Kingdom in 1990 is the basic regulation for medical waste management related policies. This law clearly stipulates the functional departments and standardized operations for medical waste management in the UK. On this basis, the United Kingdom has successively promulgated the “Hazardous Waste Regulations” (2005 Revised Edition), “Waste Management and Control Regulations” (2012), “Statutory Prudent Responsibility Regulations”, “Waste Collection and Disposal Act”, and other regulations and established a legal system covering the entire process of medical waste from generation to disposal. In 2013, various departments in the United Kingdom jointly issued the “Environment and Sustainable Health Technical Memorandum 07-01: Safe Management of Medical Waste”, which is regarded as a systematic guide for the management and disposal of medical waste. The content of this regulation covers all aspects of medical waste management in the UK, including the types of medical waste, assessment procedures, storage, transfer measures, disposal standards, technical systems, and waste management permits.

#### 4.2.3. Japan

In Japan, medical waste is classified as industrial waste (household medical waste is classified as general waste). Specifically, medical waste is divided into three categories: infectious, noninfectious, and radioactive medical waste. The “Laws Concerning the Disposal and Cleaning of Wastes” is the core regulation in the field of solid waste management in Japan, and it also proposes detailed requirements for the disposal of medical waste. Besides, for the disposal of infectious medical waste, Japan promulgated the “Guidelines for the Disposal of Infectious Waste Based on the Waste Disposal Law” in 1992 (revised in 2018). This guidance document clearly stipulates the responsible body, management standards, storage and transportation facilities, and final disposal procedures of infectious medical waste management. Japan also introduced some industry guidelines and technical standard documents related to medical waste treatment, such as “Infectious Waste Disposal Guidelines” (2009), “Infectious Waste Collection and Transportation Independent Standards”, and “Infectious Waste Incineration and Disposal Standards”. In terms of medical waste management, the Ministry of the Environment of the Japanese central government is responsible for formulating relevant policies, regulations, and technical standards, while the environmental bureau of the local government is responsible for supervision and enforcement. Under the joint action of the central government, local governments, and professional institutions, Japan has formed an effective management system for all aspects of medical waste from generation, collection, transportation, storage, treatment, and final disposal [55].

## 5. Operation Mode of Medical Waste Disposal in China

**Figure 5:**
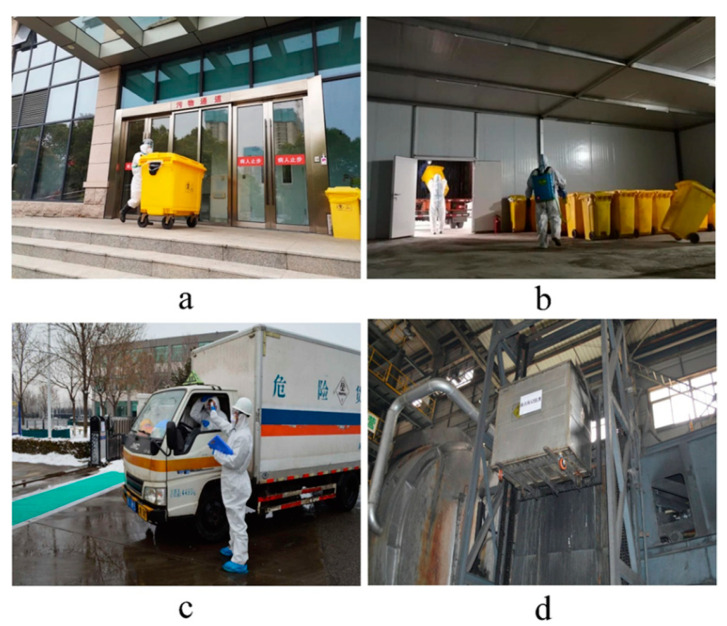
Operation mode of medical waste disposal: collection (**a**), storage (**b**), transportation (**c**), disposal (**d**). From [57,58,59].

According to existing laws and regulations, China advocates the centralized and harmless disposal of medical waste. Medical institutions have special departments or personnel responsible for the classified collection, transfer, and disposal of medical waste [56]. The management of medical waste by medical institutions must meet both the statutory requirements for medical waste management and the statutory requirements for hazardous waste management. Generally speaking, the management and disposal process of medical waste mainly includes five procedures of collection, storage, transportation, disposal, and inspection (Figure 5).

(1) Collection

Medical institutions should collect medical waste in a timely manner and adopt different collection methods for waste of different nature. The packaging container for collecting medical waste should be special packaging containers that meet the technical standards. When the outer surface of the packaging or container is contaminated with infectious waste, the contaminated area should be disinfected, or a layer of packaging should be added. The special packaging and containers for medical waste shall have obvious warning signs and warning instructions.

(2) Storage

Medical institutions should establish temporary storage facilities and equipment for medical waste and should not store medical waste in the open air. The storage of medical waste should be classified according to the characteristics of medical waste. Infectious waste, pathological wastes, noxious wastes, pharmaceutical wastes, and chemical wastes cannot be stored together, and the temporary storage time shall not exceed 2 days. Mixing medical waste into other wastes and domestic garbage is also not allowed. Storage facilities or equipment should meet environmental protection and sanitation requirements. Temporary storage facilities and equipment for medical waste shall be far away from medical areas, food processing areas, personnel activity areas, and domestic garbage storage sites. Furthermore, temporary storage facilities and equipment for medical waste should be regularly disinfected and cleaned, and hazardous waste identification signs should be set up. Hazardous waste identification signs and obvious warning signs should also be set up in the places, facilities, and equipment for storing medical waste.

(3) Transportation

Medical institutions should use special delivery vehicles with obvious warning signs and warning instructions. Medical institutions shall collect and transport medical waste to temporary storage locations in accordance with the delivery time and route determined by the unit. After the daily delivery work is completed, the delivery tools should be disinfected and cleaned in a timely manner at the designated place in the medical and health institution. Medical institutions may not discard or abandon medical waste along the way during transportation, and must take measures to prevent scattering, loss, leakage, or other measures to prevent environmental pollution.

(4) Disposal

Medical institutions should hand over medical waste to a centralized disposal unit that has obtained an operating license, but not to units or individuals that do not have business qualifications. High-risk wastes should be sterilized onsite before being handed over to a unit for centralized disposal of medical wastes. Under normal circumstances, high-risk waste needs to be sterilized by pressure steam or chemically at the production site, and then treated as infectious waste [60]. Disposable medical equipment and medical wastes that are easy to cause injury should be incinerated in time if they can be incinerated; if not, they should be disinfected and landfilled. The domestic garbage produced by infectious disease patients or suspected infectious disease patients admitted by medical institutions shall be managed and disposed of as medical waste.

(5) Check

Regular inspection of medical waste disposal is helpful to strengthen management and improve the level of medical waste disposal. Medical institutions should cooperate with relevant departments in the inspection, monitoring, investigation, and evidence collection. If medical institutions encounter emergency situations in the process of disposing of medical waste, they must promptly report to their superiors to minimize the social harm and environmental impact that may be caused by improper medical waste disposal. Medical institutions are not allowed to refuse, obstruct, or provide false materials.

## 6. Regulations and Operating Modes for Emergency Disposal of Medical Waste Related to COVID-19

### 6.1. Regulations for the Emergency Disposal of Medical Waste

**Figure 6:**
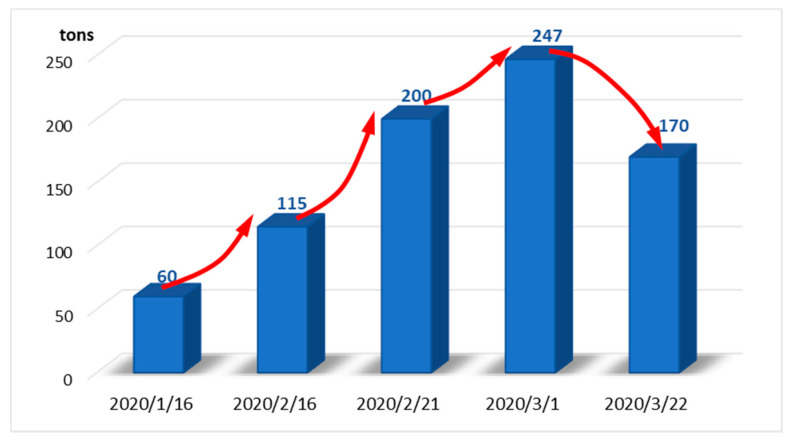
Daily output of medical waste in Wuhan. From [62].

During the COVID-19 epidemic, the number of patients increased, and the output of medical waste surged [61]. The output of medical waste in Wuhan has risen from about 40 tons per day to a peak of 247 tons (Figure 6). The disposal pressure is huge. Once these medical wastes containing the new coronavirus cannot be disposed of in a timely and safe manner, the virus will flow into the public space with the items, endangering the health and safety of the people. According to data from the Chinese Ministry of Ecology and Environment, since 20 January, a total of 256,000 tons of medical waste have been disposed of nationwide. As of April 11, the national daily disposal capacity of medical waste was 6062.4 tons, an average daily increase of 1159.6 tons compared with 4902.8 tons before the epidemic. Among them, the daily treatment capacity of medical waste in Hubei Province has increased from 180 tons before the outbreak to 667.4 tons per day, and Wuhan city has increased from 50 tons before the outbreak to 265.6 tons per day. The rapid improvement of the daily treatment capacity of medical waste is largely due to the rapid response of the government and the active response of local and medical institutions.

**Figure 7:**
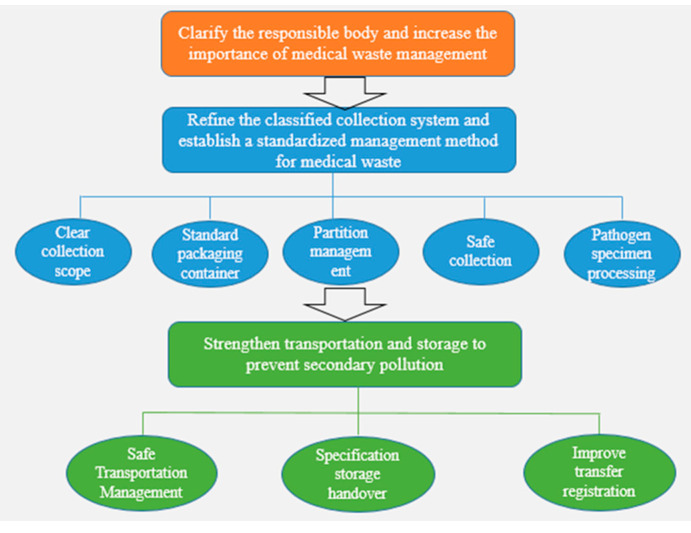
Standardized disposal of medical waste. From [63].

Hospitals all over the country are facing great pressure when disposing of medical waste, and many hospitals even have a scene of waste accumulation. Under the state of emergency, the questions of how to quickly enhance the treatment capacity of medical waste and safely dispose of medical waste have once again attracted people’s attention. At present, epidemic prevention and control is at a critical period, and whether medical waste can be handled in compliance with regulations has become an important part of combating the epidemic. Performing a good job of supervising the legal disposal of medical waste is not only a task set by the central government for governments at all levels, but also a legal requirement and a legal responsibility of government departments at all levels. The government’s quick response provides policy guarantee for the standardized disposal of epidemic medical waste. In the early stage of the outbreak, the General Office of the National Health Commission formulated and issued the “Notice on Doing a Good Job in the Management of Medical Wastes in Medical Institutions during the Pneumonia Outbreak of New Coronavirus Infection”, which clarified the strict control of the medical waste generated from the source (Figure 7).

After the outbreak, the Central Committee of the Communist Party of China issued the “Notice on Strengthening the Leadership of the Party and Providing a Strong Political Guarantee to Win the Epidemic Prevention, Control and Blocking Warfare”, and the Ministry of Ecology and Environment of the People’s Republic of China issued “About the Medical Waste Environment for the New Coronavirus Infected Pneumonia Management Notice” and other policies. On 3 March 2020, the Ministry of Ecology and Environment of the People’s Republic of China issued the “Guiding Opinions on Coordinating the Prevention and Control of Epidemic Situation and the Ecological and Environmental Protection of Economic and Social Development”. It requires 100% coverage of environmental supervision and services of all medical institutions and facilities across the country, and 100% implementation of timely and effective collection, transshipment, treatment, and disposal of medical waste and medical wastewater. It emphasizes the focus on designated medical places, comprehensively understanding the production, collection, transshipment, storage, treatment, and disposal of epidemic medical wastes in various places to ensure the collection and disposal of all waste. Various local governments have also actively formulated and issued outbreak response plans and measures and have set clear requirements for the management and control of medical waste collection and disposal (Table 4).

### 6.2. Operation Mode for Emergency Treatment of Medical Waste

**Figure 8:**
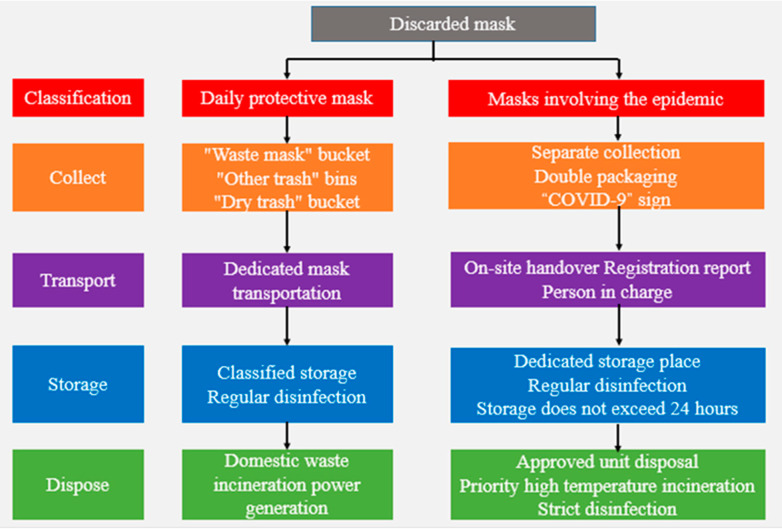
Medical waste disposal process system. From [64].

The medical waste of the COVID-19 epidemic is infectious medical waste. According to the “National List of Hazardous Wastes”, the waste category is HW01 medical waste, and the waste code is 831-001-01. During the epidemic, the reasonable disposal of medical waste has become an important link to prevent secondary pollution. In the research and practice of the government and medical institutions at all levels, a comprehensive, refined, and standardized medical treatment process system has been formed. Take the discarded mask as an example (Figure 8):

(1) Collection

Medical institutions should give priority to the collection of infectious medical waste generated during the epidemic prevention and control, and carry out a good job of sorting, collecting, and storing the medical waste from epidemic and nonepidemic medical waste. Double-layer packaging bags are used to contain medical waste, which are layered and sealed. When sorting and collecting disposable articles such as disposable gowns and protective clothing, squeezing is strictly prohibited. Each packaging bag and sharps box should be affixed with a Chinese label, and the label should be marked with “COVID-19”.

(2) Storage

The medical waste generated in the potentially contaminated area and the contaminated area of patients and suspected patients should be sprayed and disinfected on the surface of the packaging bag or covered with a layer of medical waste packaging bag before leaving the contaminated area. The storage place should be sterilized according to the method and frequency required by the competent health department, and the temporary storage time should not exceed 24 h.

(3) Transportation

Under emergency conditions, in addition to the use of special transport vehicles, the transportation of infectious medical waste generated during the epidemic prevention and control process can also use vehicles that have been temporarily modified with reference to the requirements of medical waste transport vehicles. The medical waste transfer process can use electronic transfer orders or paper orders according to local conditions. The medical waste generation department, delivery personnel, temporary storage staff, and the transfer personnel of the disposal unit should register and hand over each layer and explain that it originated from a COVID-19 patient or suspected patient. In addition to the legally required source, type, weight or quantity, delivery time, final destination, and signature of the handler, the content of medical registration should specifically indicate the words “COVID-19”. The registration information is kept for 3 years. Medical waste should be transferred to disposal facilities within 48 h. Special vehicles transport infectious medical wastes generated during the prevention and treatment of pneumonia epidemics separately, and do not mix with other medical wastes. The transfer form should be filled out separately to other medical waste and a ledger established.

(4) Disposal

During the epidemic, the disposal of medical waste mainly includes the following four aspects:

First, an emergency plan for medical waste disposal should be started. Units engaged in the centralized disposal of medical wastes shall apply to the municipal environmental protection department for the hazardous waste business license, otherwise they shall not engage in activities related to the centralized disposal of medical wastes. During emergency disposal, medical institutions shall give priority to the use of centralized medical waste disposal facilities within their administrative area. When the existing disposal capacity in the area cannot meet the needs of emergency disposal, the emergency plan should be immediately initiated, and the emergency disposal facilities included in the emergency disposal resource list should dispose of the medical waste and carry out designated management. Alternatively, the medical institution may transfer the medical waste to a centralized medical waste disposal facility in a nearby area for disposal. If the conditions for centralized disposal are not met, medical waste may be incinerated and disposed in situ according to the plan determined by the local people’s government.

Second, medical waste disposal facilities should be used for emergency disposal of medical waste. During emergency disposal, when medical institutions adopt mobile medical waste disposal facilities for emergency disposal of medical waste, they can be exempted from environmental impact assessment, medical waste business license, and other procedures. However, medical institutions should set up locations reasonably to avoid environmentally sensitive areas such as drinking water source protection areas. Suppliers of mobile medical waste disposal facilities shall ensure that the effects of medical waste disposal meet the requirements of relevant standards and technical specifications.

Third, infectious medical waste should be managed separately from other medical waste. Medical institutions should try to classify and manage infectious medical waste and other medical waste generated during the prevention and treatment of pneumonia. Centralized medical waste disposal facilities and mobile medical waste disposal facilities shall be given priority for the treatment of infectious medical waste generated during the prevention and treatment of pneumonia. Other medical waste can be diverted to other emergency disposal facilities for disposal.

Fourth, considering the rapid infectiousness of COVID-19, high-temperature incineration is a priority for medical waste. Each region can choose the emergency treatment method for medical waste of pneumonia according to the actual situation and local conditions. Medical wastes with pneumonia should be treated with high-temperature incineration. In addition, no incineration methods, such as high-temperature steam sterilization, microwave sterilization, and chemical sterilization, can also be used. The disposal of medical waste with pneumonia epidemic in other ways should pay attention to ensure the disposal effect. The temperature in the cement kiln is about 1400 °C, which can completely extinguish the new coronavirus. It has the characteristics of high disposal efficiency, no secondary pollution, high safety, and resource utilization, and it is one of the effective methods of COVID-19 medical waste treatment.

(5) Check

During the COVID-19 pandemic, medical institutions must register while collecting and transferring medical waste. Relevant responsible personnel should truthfully record the source, type, weight or quantity of medical waste, handover time, final destination, and signature of the person in charge, with special indication of “new coronavirus-infected pneumonia” or “COVID-19”. The registration information needs to be kept for 3 years for inspection.

## 7. Conclusions

The diagnosis and treatment of COVID-19 patients not only produced conventional medical waste, but also produced contaminated protective clothing, masks, gloves, and other protective equipment [65]. In addition, there are patients’ household garbage. This has led to a surge in the total amount of medical waste and increased pressure on medical waste disposal. Unlike ordinary wastes and garbage, medical wastes that are untreated or incompletely treated will not only cause environmental pollution, but also directly or indirectly cause infections and endanger people health [66,67].

This article draws the following conclusions through research:

(1) During the COVID-19 epidemic, the number of patients increased, and the output of medical waste surged. The output of medical waste in Wuhan has risen from about 40 tons per day to a peak of 247 tons. According to data from the Chinese Ministry of Ecology and Environment, since 20 January, a total of 256,000 tons of medical waste have been disposed of nationwide. As of April 11, the national daily disposal capacity of medical waste was 6062.4 tons, an average daily increase of 1159.6 tons compared with 4902.8 tons before the epidemic.

During the COVID-19 epidemic, the daily generation of China’s medical waste has increased significantly, placing a severe test on China’s medical waste disposal system. Faced with difficulties, the Chinese government has formulated a policy for medical waste management and a response plan for the epidemic, which provides policy guarantee for the standardized disposal of epidemic medical waste [68]. In addition, the government and medical institutions at all levels have formed a comprehensive, refined, and standardized medical treatment process system during research and practice. China has increased the capacity of medical waste disposal in various places by constructing new centralized disposal centers and adding mobile disposal facilities. Therefore, effective response measures across the country ensure the proper disposal of medical waste.

(2) The “Law of the People’s Republic of China on Prevention and Control of Infectious Diseases” clearly stipulates the disposal of medical waste, supervision and management, and legal accountability. Since then, laws and regulations have been issued one after another, which fully illustrates the importance the Chinese state, government, and people attach to the management of medical waste. After years of practical discussion and theoretical research, a relatively complete medical waste management legal system has gradually been formed. The continuous improvement of the medical waste management system can not only effectively block the spread of pathogenic microorganisms and reduce hospital infection and social pollution, but also provide institutional guarantee for the improvement of environmental quality and the healthy development of ecology. In addition, standardized management of medical waste has played a good role in reducing the economic cost of treating medical waste, reducing the government’s economic burden, and saving environmental resources, so as to achieve a win–win situation for social, economic, and environmental benefits.

(3) On the one hand, the COVID-19 outbreak improved China’s medical waste disposal capacity; on the other hand, the sudden emergence of public health emergencies also exposed China’s insufficient medical waste management [69]. First, China’s laws and regulations are not perfect, and the ability to monitor medical waste is insufficient. There are still many legislative blind spots in the existing laws and regulations regarding the disposal of medical waste. For example, there is no effective system that can be followed for the classification of medical waste; there is no allocation of responsibilities to producers. Second, there is a gap in the level of medical waste treatment in various regions of China. With the improvement of urbanization level and the increase of urban population, the urban medical waste disposal capacity and infrastructure construction urgently need to be strengthened. Third, the sudden outbreak of COVID-19 has exposed China’s medical waste disposal capacity to be insufficiently prepared to respond to emergencies. At present, the centralized disposal capacity of medical waste in many cities in China does not match the growth rate of medical waste.

## Figures and Tables

**Table 1:** Classification of medical waste.

Category	Common Ingredients
Infectious waste	Blood, body fluids, excreta, or wastes of patients with the risk of causing the spread of infectious diseases.
Pathological waste	Abandoned human tissues, corpses of medical laboratory animals, etc.
Noxious waste	Injurious waste refers to discarded medical sharps that can puncture or cut the human body, including medical needles, scalpels, scalpels, glass test tubes, etc.
Pharmaceutical waste	Expired, eliminated, deteriorated, or contaminated waste medicine.
Chemical waste	Waste chemicals that are toxic, corrosive, flammable, and explosive.

From [28].

**Table 2:** Medical waste disposal technology.

	Common Technology	Technical Characteristics	Applicable Scope
Incineration	Rotary kiln burning	It has large processing capacity, wide application range, full combustion, and can run stably for a long time.	Infectious waste, pathological waste, injury waste, pharmaceutical waste, chemical waste
	Pyrolysis incineration	It has a small discharge volume and a high pyrolysis rate.	
Disinfection	High temperature steam disposal	It has small processing capacity, low operating cost, less secondary pollution, low operation and management difficulty, and is suitable for intermittent operation.	Infectious waste, injury waste, part of pathological waste
	Chemical disinfection		
	Microwave disinfection		

**Table 3:** Laws and regulations.

Year	Laws and Regulations	Content
1989	“Law of the People’s Republic of China on Prevention and Treatment of Infectious Diseases”	It clearly stipulates the disposal of medical waste, supervision and management, and legal accountability.
1996	“Environmental Sanitation Standards for Medical Waste Incineration”	It stipulates the standard values of medical waste incineration and detection methods.
1996	“Law of the People’s Republic of China on the Prevention and Control of Solid Waste Pollution”	It provides for the prevention and control of hazardous waste pollution.
2003	“Medical Waste Management Regulations”	It strengthens the safety management of medical waste.
2003	“Standard for Pollution Control on Hazardous Waste Storage”	It is the latest national unified regulations on the management of medical waste in China.
2003	“Administrative Penalties for Medical Waste Management”	It provides for administrative penalties for violations of medical waste management regulations.
2003	“Administrative Measures on Medical Waste of Medical institutions”	It regulates the management of medical waste by medical institutions.
2003	“Emergency Regulations for Emergencies”	It proposes effective prevention, timely control, and elimination of the hazards of public health emergencies.
2003	“Classified catalogue of medical wastes”	It has a unified classification of medical waste.
2003	“Technical Specifications for Centralized Disposal of Medical Waste”	It clearly stipulates the temporary storage, transportation, handover, and disposal of medical waste.
2003	“Technical Requirements for Medical Waste Transfer Vehicles”	It stipulates special requirements for medical waste transfer vehicles.
2003	“Environmental Sanitation Standards for Medical Waste Incineration”	It stipulates the standard values and monitoring methods for the environmental sanitation of medical waste incineration.
2015	“Environmental Protection Law” (Revision)	It has newly added medical waste in the traditional pollution category.

**Table 4:** New policies and notices.

No.	Issuer	Release Time	Policy/Notice
1	Ministry of Ecology and Environment of the People’s Republic of China	21 January 2020	“Notice on Doing a Good Job in Environmental Management of New Coronavirus Infected Pneumonia Outbreak Medical Wastes”
2	National Health Commission of the People’s Republic of China	25 January 2020	“Notice on strengthening community prevention and control of pneumonia outbreaks of new coronavirus infection”
3	National Health Commission of the People’s Republic of China	26 January 2020	“Notice on strengthening the prevention and control of pneumonia outbreaks of new coronavirus infections in primary medical institutions”
4	National Health Commission of the People’s Republic of China	27 January 2020	“New Coronavirus Infected Pneumonia Case Transfer Work Program” (Trial)
5	Ministry of Ecology and Environment of the People’s Republic of China	28 January 2020	“New Coronavirus Infected Pneumonia Outbreak Medical Waste Emergency Management and Technical Guideline” (Trial)
6	National Health Commission of the People’s Republic of China	28 January 2020	“Notice on the management of medical waste in medical institutions during the pneumonia outbreak of new coronavirus infection”
7	National Health Commission of the People’s Republic of China	28 January 2020	“Notice on the management of medical waste during the outbreak of pneumonia caused by new coronavirus”
8	National Health Commission of the People’s Republic of China	28 January 2020	“Notice on further strengthening the medical treatment of pneumonia caused by new coronavirus infection in counties”
9	Ministry of Ecology and Environment of the People’s Republic of China	1 February 2020	“Notice on Doing Well the Supervision Work on Medical Sewage and Urban Sewage of New Coronavirus Infected Pneumonia”
10	Ministry of Ecology and Environment of the People’s Republic of China, National Health Commission of the People’s Republic of China, etc.	24 February 2020	“Work plan for comprehensive treatment of waste in medical institutions”
11	Department of Ecology Environment of Hubei Province	22 January 2020	“Urgent notice on how to effectively deal with the pneumonia epidemic of new coronavirus infection and strengthen the environmental management of medical waste”
12	Department of Ecology and Environment of Sichuan Province	29 January 2020	“Urgent notice on the establishment of emergency facilities for emergency disposal of medical waste in the province with new coronavirus infection pneumonia”
13	Department of Ecology and Environment of Hunan Province	29 January 2020	“Urgent notice on practically strengthening the management of medical waste water and special garbage of pneumonia epidemic infected with new coronavirus”
14	Department of Ecology and Environment of Shandong Province	30 January 2020	“Notice on the collection, transportation and disposal of special hazardous waste such as waste masks during the epidemic”
15	Department of Ecology and Environment of Anhui Province	30 January 2020	“Notice regarding the issuance of management and technical guidelines for emergency treatment of medical wastes from pneumonia outbreaks of new coronavirus infection (for trial implementation)”
16	Department of Ecology and Environment of Fujian Province	30 January 2020	“New Crown Pneumonia Epidemic Medical Waste Emergency Disposal Enterprise Operation Management Regulations”
17	Department of Ecology and Environment of Jilin Province	1 February 2020	“Guidance on further improving the treatment of medical waste and discarded masks during the prevention and control of new coronavirus infection pneumonia”
18	Zhejiang Province	3 February 2020	“Notice on clarifying the work requirements of each link of environmental management of medical waste of pneumonia epidemic infected with new coronavirus”
19	Guangdong Province	29 January 2020	“Notice on strengthening the management of discarded masks and doing a good job in the prevention and control of new coronavirus infection pneumonia”

## Data Availability

Not applicable.
